# Inverse Association Between Dietary Niacin Intake and Prevalence of Depressive Symptoms in Adults With Metabolic‐Associated Fatty Liver Disease: A Cross‐Sectional Study

**DOI:** 10.1002/fsn3.71940

**Published:** 2026-06-19

**Authors:** Xiaoxian Yang, Haiyi Yan, Xueying Qin, Yan Chen

**Affiliations:** ^1^ Xiyuan Hospital of China Academy of Chinese Medical Sciences Beijing China; ^2^ First Clinical Medical College, Nanjing University of Chinese Medicine Nanjing China; ^3^ The First Teaching Hospital of Tianjin University of Traditional Chinese Medicine Tianjin China

**Keywords:** depression, fatty liver, MAFLD, NHANES, niacin intake

## Abstract

Depression and fatty liver disease exhibit a close bidirectional relationship, and depressed patients with fatty liver disease represent a clinically important subgroup. Although niacin intake has been shown to ameliorate fatty liver disease, its effects on depression remain inconsistent. This cross‐sectional study used data from the National Health and Nutrition Examination Survey (NHANES) 2017–2020 to investigate the association between niacin intake and depression in individuals with metabolic dysfunction‐associated fatty liver disease (MAFLD). Multivariate logistic regression was applied to evaluate the linear association, while smooth curve fitting and threshold effect analysis were performed to characterize nonlinear relationships. Subgroup analyses and interaction tests were conducted to assess the robustness of the findings. A nonlinear association was observed between niacin intake and depression in MAFLD patients. Threshold analysis identified an inflection point at approximately 10.24 mg/day, below which a significant inverse association was found (OR 0.80; 95% CI: 0.68–0.93), whereas no significant relationship was detected above this threshold. Subgroup analyses and interaction tests consistently confirmed the robustness of these findings. In conclusion, moderate niacin intake is associated with a lower risk of depression in patients with MAFLD, and the identified threshold of approximately 10.24 mg/day may serve as a reference value for future research.

## Graphical abstract

This cross‐sectional study included adults with MAFLD. Logistic regression and smooth curve fitting were used to evaluate the association between dietary and supplemental niacin intake (mg/day) and depression (PHQ‐9 score ≥ 10). The results indicate that moderate niacin intake may reduce the risk of depression in individuals with MAFLD, with a potential dual mechanism involving hepatic lipid metabolism and neuroemotional regulation. These findings suggest that ensuring moderate dietary niacin intake is of public health importance for mental health in MAFLD patients.

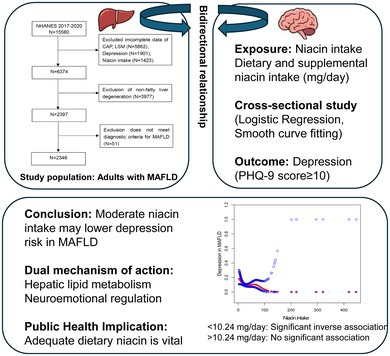

## 1 Introduction

Depression is a common psychiatric disorder characterized primarily by persistent low mood, pessimism, poor sleep quality, diminished life satisfaction, and a markedly elevated risk of suicide (Krishnan and Nestler 2008). Reports indicate that approximately 17.3 million adults in the United States experienced at least one major depressive episode in 2017, corresponding to a prevalence rate of 7.1% (Liu et al. 2020). By 2020, the global incidence of depression had risen by 28% (Zhao et al. 2023), affecting nearly 300 million people worldwide (Malhi and Mann 2018). The onset of depression severely compromises quality of life, threatens both physical and mental health, and creates a substantial economic burden, positioning it as a major public health concern (Chisholm et al. 2016). Fatty liver disease is a rapidly progressing chronic liver condition. In 2020, an international expert panel introduced new diagnostic criteria for MAFLD. Unlike nonalcoholic fatty liver disease (NAFLD), MAFLD incorporates additional markers of metabolic dysregulation beyond simple hepatic steatosis, independent of alcohol consumption (Wong and Lazarus 2021).

Multiple evidence‐based analyses have consistently demonstrated that the pooled prevalence of depression is significantly elevated among patients with fatty liver disease, indicating a strong link between the two conditions (S. Li et al. 2024; Mou and Yao 2025; Shea et al. 2024). Mendelian randomization studies have further supported a causal relationship, revealing a positive causal association between depression and MAFLD, with major depression identified as a potential causal risk factor for fatty liver disease (Liang et al. 2024; Su et al. 2025). Clinical studies have directly observed that the prevalence of depressive symptoms in MAFLD patients reaches as high as 75%, markedly higher than the 16.4% observed in control groups (Mostafa et al. 2024). Moreover, numerous studies have revealed multidimensional shared pathways between fatty liver disease and depression, encompassing genetic, metabolic, inflammatory, and environmental factors (Guo et al. 2025; Meroni et al. 2025; Sampada et al. 2025). Collectively, these findings point to a bidirectional association between fatty liver disease and depression, highlighting that MAFLD patients with depression constitute a clinically meaningful subgroup that deserves special attention.

Substantial evidence suggests that severe B‐vitamin deficiencies are associated with a higher incidence of psychiatric disorders (D. Li et al. 2022; Mahdavifar et al. 2021). Niacin (vitamin B_3_), an essential B vitamin, plays critical roles in central nervous system function, neuronal development, and lipid metabolism regulation (D'Andrea et al. 2019; Sarkar et al. 2020). Notably, niacin exhibits a unique dual mechanism of action. On one hand, it reduces hepatic triglyceride synthesis and modulates nicotinamide Nmethyltransferase (NNMT) to influence NAD^+^ metabolism, thereby participating in the pathogenesis of hepatic steatosis via the gut–liver axis (Hu et al. 2012; Pirinen et al. 2020; Zhang et al. 2025). On the other hand, niacin inhibits neuroinflammation by activating the GPR109A receptor and alleviates endothelial oxidative stress by suppressing reactive oxygen species production (Ganji et al. 2014, 2009; Ibrahim et al. 2022). This dual capacity to simultaneously target hepatic lipid metabolism and neuroinflammatory pathways gives niacin a potential therapeutic value in individuals with MAFLD and comorbid depression—a benefit that other B vitamins are unlikely to provide. In contrast, the antidepressant effects of other B vitamins (e.g., B_1_, B_2_, B_6_, and B_12_) are achieved indirectly, primarily by lowering homocysteine levels, without directly regulating hepatic lipid metabolism (Wu et al. 2022).

Although the efficacy of niacin against fatty liver disease has been well established, its effects on depression remain controversial (Mishra et al. 2009). This evidence raises an important and previously underexplored question: Can niacin provide therapeutic benefits to depressed individuals within the MAFLD population?

## 2 Methods

### 2.1 Survey Description

This cross‐sectional study used data from the National Health and Nutrition Examination Survey (NHANES), a nationally representative program conducted by the National Center for Health Statistics (NCHS) to evaluate the health and nutritional status of the US population. The survey employed a complex, multistage probability sampling design to ensure optimal population representativeness. The NHANES study protocol was approved by the NCHS Research Ethics Review Board, and obtained written informed consent from all participants. Complete NHANES documentation and datasets are publicly available at https://www.cdc.gov/nchs/nhanes/.

### 2.2 Study Population

**FIGURE 1:**
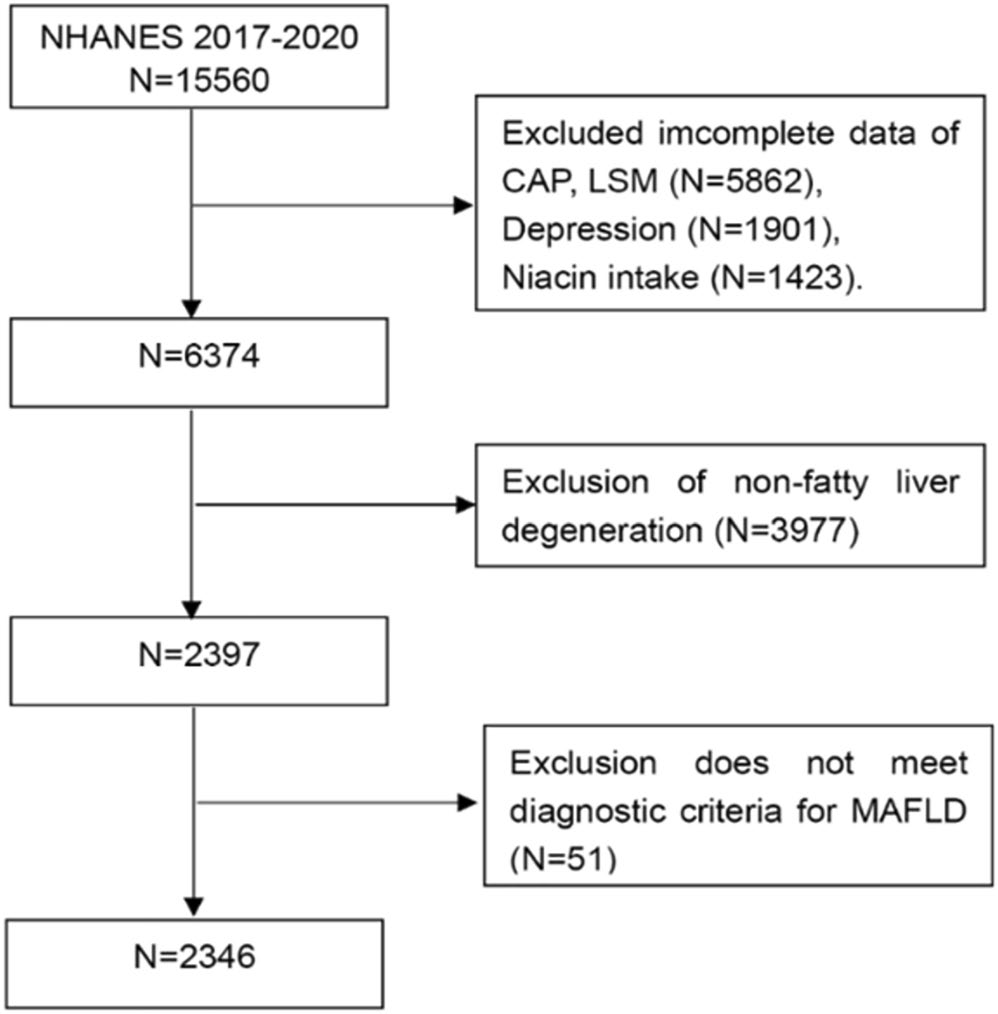
Flowchart of the sample selection from NHANES 2017–2020.

We used data from the 2017–2020 NHANES cross‐sectional survey, which included liver ultrasound transient elastography (FibroScan) measurements. From an initial cohort of 15,560 adult participants (aged ≥ 18 years), we sequentially excluded: (1) 5862 individuals with missing controlled attenuation parameter (CAP) or liver stiffness measurement (LSM) data; (2) 1901 with incomplete depression status information; and (3) 1423 lacking niacin intake data. This yielded 2397 participants with hepatic steatosis. After applying the most recent MAFLD diagnostic criteria, we further excluded 51 ineligible subjects, resulting in a final analytical sample of 2346 participants (Figure 1).

### 2.3 Definition

#### 2.3.1 Assessment of Dietary Niacin Intake

Dietary and supplemental niacin intake data were collected through two 24‐h recall interviews in NHANES. The first interview was conducted in person at the Mobile Examination Center (MEC), followed by a telephone interview 3–10 days later. Total niacin intake was calculated as the mean of the two dietary recalls combined with the two supplemental intake measurements.

#### 2.3.2 Definition of MAFLD

The newly established MAFLD diagnostic criteria require hepatic steatosis accompanied by at least one of the following metabolic features: (1) overweight/obesity (BMI ≥ 25 kg/m^2^), (2) type 2 diabetes mellitus (diagnosed by physician confirmation, abnormal glucose tolerance test, or use of glucose‐lowering medication), or (3) the presence of ≥ 2 other metabolic risk abnormalities (Eslam et al. 2020). In this study, we operationally defined: hepatic steatosis as CAP ≥ 285 dB/m; clinically significant fibrosis (CSF; stage ≥ F2) as LSM ≥ 8.6 kPa (Kim et al. 2023).

#### 2.3.3 Definition of Depression

The Patient Health Questionnaire‐9 (PHQ‐9) is a self‐administered screening tool developed based on the diagnostic criteria of the Diagnostic and Statistical Manual of Mental Disorders (DSM) to assess the presence and severity of depressive symptoms. It evaluates the frequency of depressive symptoms over the preceding 2 weeks and has become one of the most widely implemented depression screening instruments worldwide (Kroenke 2021). According to DSM‐IV criteria, a total score of ≥ 10 is considered clinically relevant depression (Chunnan et al. 2022; Kroenke et al. 2001).

### 2.4 Selection of Covariates

The study covariates included demographic characteristics (gender, age, race [non‐Hispanic white, non‐Hispanic black, Mexican American, other], education level [< high school, high school graduate, > high school], and economic status categorized by poverty‐income ratio [low income: PIR < 1.3; middle income: 1.3 ≤ PIR < 3.5; high income: PIR ≥ 3.5]), behavioral and anthropometric measures (smoking status [≥ 100 lifetime cigarettes], BMI categories [normal weight: 18.5 ≤ BMI < 25 kg/m^2^; overweight: 25 ≤ BMI < 30; obese: BMI ≥ 30], waist circumference, and total daily energy intake), and physician‐diagnosed chronic conditions (hepatitis B/C, diabetes mellitus, hypertension, and severe cardiovascular disease [CVD] including coronary heart disease, myocardial infarction, congestive heart failure, angina pectoris, and stroke).

### 2.5 Statistical Analysis

All statistical analyses were conducted using R software (version 4.1.3) and EmpowerStats (version 2.0) for data processing and visualization. All analyses, including spline fitting and threshold effect analysis, applied the sampling weight “WTDR2D” provided by NHANES. Continuous variables are presented as mean ± standard deviation (SD), and categorical variables as percentages (%). Multivariable logistic regression models were constructed to examine the association between niacin intake and depression in MAFLD patients, with results expressed as adjusted odds ratios (ORs) with 95% confidence intervals (CIs). Three sequential models were implemented: Model 1 (unadjusted), Model 2 (adjusted for gender, age, and race), and Model 3 (fully adjusted for all covariates). Sensitivity analyses included quartile‐based categorization of niacin intake. Nonlinear relationships were evaluated using smooth curve fitting, and threshold effect analysis identified significant breakpoints. Subgroup analyses and interaction tests assessed effect modification across population strata. All statistical tests were two‐tailed, with *p* < 0.05 considered statistically significant.

Missing covariate data were handled as follows. For categorical covariates, missing values were grouped into a separate category during analysis to avoid interference with complete data. For continuous covariates, missing values were imputed using the mean or median. Alternatively, when appropriate, continuous variables were converted to categorical variables, with missing values assigned to a dedicated group before statistical analysis.

## 3 Results

### 3.1 Baseline Characteristics of Participants

The study included 2346 eligible participants with MAFLD (mean age 51.24 ± 16.14 years; 44.14% female, 55.86% male). As shown in Table 1, non‐Hispanic Whites constituted the predominant ethnic group (64.67%), and depressive symptoms were present in 9.26% of the MAFLD population. Higher niacin intake was significantly associated (*p* < 0.05) with several characteristics: male predominance, married/cohabiting status, higher educational attainment, and higher socioeconomic status. Compared to those with lower niacin consumption, individuals with higher niacin intake also had lower smoking rates, lower prevalence of CVD, diabetes, hepatitis B, and CSF, as well as lower depression rates, greater waist circumference, and higher total energy intake.

**TABLE 1:** Basic characteristics of the study population.

Characteristics	Overall (*n* = 2346)	Q1 (*n* = 587)	Q2 (*n* = 586)	Q3 (*n* = 586)	Q4 (*n* = 587)	*p*
Age, years	51.24 ± 16.14	52.05 ± 16.39	49.90 ± 16.11	49.61 ± 16.55	53.53 ± 15.33	< 0.0001
Gender, %						
Male	55.86	33.83	53.93	61.19	68.32	< 0.0001
Female	44.14	66.17	46.07	38.81	31.68	
Race, %						
Non‐Hispanic white	64.67	58.82	61.72	64.99	71.32	0.0003
Non‐Hispanic black	9.06	12.99	10.69	7.82	5.93	
Mexican American	11.04	11.28	11.30	11.96	9.65	
Others	15.23	16.92	16.29	15.23	13.09	
Marital status, %						
Married/Living with Partner	67.08	59.44	66.89	68.28	72.02	< 0.0001
Widowed/Divorced/Separated	17.51	23.95	17.72	12.96	16.78	
Never married	14.22	14.66	14.26	17.13	10.93	
Education level, %						
Less than high school	9.52	13.78	11.93	7.71	6.06	< 0.0001
High school	29.27	36.76	30.92	25.45	25.76	
Above high school	59.98	47.52	55.88	65.21	67.92	
Others	1.23	1.94	1.28	1.64	0.27	
Socioeconomic status, %						
Low income	10.84	16.84	9.67	9.63	8.42	< 0.0001
Middle income	45.91	49.48	51.04	43.11	42.03	
High income	34.19	25.42	30.09	37.96	40.33	
Smoking status, %						
Yes	45.55	46.53	39.86	48.05	46.77	0.0246
No	54.45	53.47	60.14	51.95	53.23	
CVD, %						
Yes	12.19	13.60	11.00	8.84	15.27	0.0012
No	86.58	84.42	87.77	89.53	84.47	
Hypertension, %						
Yes	54.65	55.59	55.05	54.45	53.80	0.9370
No	45.35	44.41	44.95	45.55	46.20	
Diabetes, %						
Yes	20.48	22.29	17.57	19.42	22.46	0.0036
No	76.06	71.82	79.40	78.45	74.17	
Borderline	3.45	5.89	3.02	2.13	3.36	
Hepatitis B, %						
Yes	1.15	0.60	1.39	0.80	1.68	0.0433
No	98.50	99.32	97.63	99.15	98.03	
Hepatitis C, %						
Yes	1.73	1.87	1.63	0.88	2.52	0.3105
No	98.14	98.04	98.37	99.00	97.21	
CSF, %						
Yes (LSM ≥ 8.6)	17.74	20.15	12.46	16.78	21.04	0.0005
No (LSM < 8.6)	82.26	79.85	87.54	83.22	78.96	
Depression, %						
Yes	9.26	13.62	9.35	7.73	7.34	0.0012
No	90.74	86.38	90.65	92.27	92.66	
BMI (kg/m^2^), %						
Normal weight	3.24	4.40	2.48	3.15	3.18	0.2828
Overweight	24.93	21.21	25.48	26.74	25.28	
Obese	71.83	74.39	72.04	70.11	71.54	
WC, cm	113.07 ± 14.97	112.04 ± 14.90	112.38 ± 13.96	112.80 ± 14.65	114.64 ± 15.92	0.0123
Total energy, kcal	2131.05 ± 797.64	1434.35 ± 491.28	1956.52 ± 513.05	2345.67 ± 687.30	2580.37 ± 872.36	< 0.0001

*Note:* Mean +/− SD for continuous variables: *p* value was calculated by weighted linear regression model. % for categorical variables: *p* value was calculated by weighted chi‐square test.

Abbreviations: CSF, Clinically significant fibrosis; CVD, cardiovascular disease; LSM, liver stiffness measurement; WC, waist circumference.

### 3.2 Association of Niacin Intake With Depression Among MAFLD Patients

Table 2 presents the association between niacin intake and depression in MAFLD patients. Our analyses showed a significant inverse relationship in both Model 1 (unadjusted) and Model 2 (partially adjusted), with each 1‐unit increase in niacin intake corresponding to a 1% reduction in depression risk (OR [95% CI]: 0.99 [0.98, 0.99]). However, this association became nonsignificant in the fully adjusted Model 3. When niacin intake was analyzed as a categorical variable (quartiles), the highest quartile showed a significant inverse association with depression risk in the fully adjusted model (OR [95% CI]: 0.61 [0.38, 0.99]). Given the exceptionally wide range of Q4 (35.71–629.72), which may have included extreme high‐dose outliers, we performed a sensitivity analysis by excluding six extreme values (> 500 mg/day) to minimize potential bias. After this exclusion, the association in Q4 became nonsignificant in the fully adjusted model (OR [95% CI]: 0.62 [0.38, 1.01]) (Table 3).

**TABLE 2:** Association between niacin intake and depression.

Quartiles	OR (95% CI)^a^		
	Crude model (Model 1)^b^	Minimally adjusted model (Model 2)^c^	Fully adjusted model (Model 3)^d^
Continuous	0.99 (0.98, 0.99)	0.99 (0.98, 0.99)	0.99 (0.98, 1.00)
Categories			
Q1 (2.52–17.96)	Reference	Reference	Reference
Q2 (17.96–25.08)	0.64 (0.44, 0.92)	0.69 (0.47, 0.99)	0.67 (0.45, 1.02)
Q3 (25.09–35.60)	0.70 (0.49, 1.01)	0.77 (0.53, 1.12)	0.70 (0.45, 1.08)
Q4 (35.71–629.72)	0.59 (0.41, 0.87)	0.67 (0.45, 0.99)	0.61 (0.38, 0.99)
*p* for trend	0.0205	0.0973	0.0988

*Note:* In sensitivity analysis, niacin intake was converted from a continuous variable to a categorical variable (quartile).

^a^ OR (95% CI): odds ratio (95% confidence interval).

^b^ Model 1 adjust for None.

^c^ Model 2 adjusts for Gender, Age, and Race.

^d^ Model 3 adjust for Gender, Age, Race, Marital status, Education level, Socioeconomic status, Smoking status, CVD, Hypertension, Diabetes, Hepatitis B, Hepatitis C, CSF, BMI, WC, and Total energy.

**TABLE 3:** Association between niacin intake and depression (excluding extreme values).

Quartile	OR (95% CI)		
	Crude model (Model 1)	Minimally adjusted model (Model 2)	Fully adjusted model (Model 3)
Continuous	0.99 (0.98, 0.99)	0.99 (0.98, 0.99)	0.99 (0.98, 1.00)
Categories			
Q1 (2.52–17.92)	Reference	Reference	Reference
Q2 (17.93–25.04)	0.66 (0.45, 0.95)	0.71 (0.49, 1.03)	0.69 (0.46, 1.04)
Q3 (25.05–35.55)	0.71 (0.50, 1.02)	0.78 (0.54, 1.13)	0.72 (0.46, 1.11)
Q4 (35.56–445.34)	0.60 (0.41, 0.88)	0.68 (0.46, 1.01)	0.62 (0.38, 1.01)
*P* for trend	0.0228	0.1053	0.1072

**FIGURE 2:**
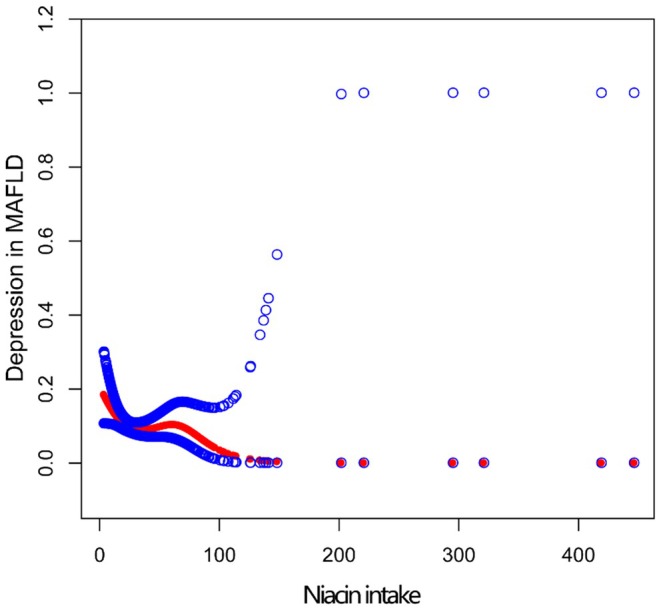
Smoothed curve fit plot.

The nonsignificant trend test (*p* for trend = 0.0988) suggested a potential nonlinear association between the variables. Consequently, we performed smooth curve fitting analysis, which revealed a nonlinear relationship between niacin intake and depression risk in MAFLD patients (Figure 2). Threshold analysis identified an inflection point at approximately 10.24 mg/day. Below this threshold, each 1‐unit increase in niacin intake was associated with a 21% reduction in depression risk (OR [95% CI]: 0.79 [0.68, 0.92]), whereas no significant association was observed above the threshold (Table 4).

**TABLE 4:** Threshold effect analysis.

	OR (95% CI), *p*
Model 1^a^	0.99 (0.98, 1.00)
Model 2^b^	
Breakpoint (K)	10.24
OR1 (< 10.24)	0.79 (0.68, 0.92)
OR2 (> 10.24)	0.99 (0.98, 1.00)
Log likelihood ratio	0.006

^a^ Model 1: Standard linear model.

^b^ Model 2: Two‐piecewise linear model. Adjust for all variables.

To validate the reliability of the approximately 10.24 mg/day threshold, we stratified the data into two subgroups using this cutoff value. The results showed that when niacin intake was below approximately 10.24 mg/day, higher intake was inversely associated with depression risk in MAFLD patients, while no significant association was observed above this threshold—findings consistent with the smooth curve fitting results (Table 5).

**TABLE 5:** Stratified analysis of niacin intake.

Niacin intake (mg/day)	OR (95% CI) Depression in MAFLD	*p* for interaction
< 10.24	0.70 (0.54, 0.90)	0.0058
≥ 10.24	0.99 (0.98, 1.00)	

## 4 Subgroup Analysis

To assess potential effect modification by baseline characteristics, we conducted stratified analyses and interaction tests across subgroups defined by gender, age, race, diabetes, CVD, hepatitis B status, and clinically significant fibrosis (CSF). The analysis revealed no significant effect modification (all *p* for interaction > 0.05), indicating consistent associations between niacin intake and depression in MAFLD patients regardless of these covariates. Notably, the sample size of patients with hepatitis B was small; therefore, this result should be interpreted with caution (Table 6).

**TABLE 6:** Subgroup analysis by baseline characteristics.

	OR (95% CI) Depression in MAFLD	*p* for interaction
Gender		
Male	0.99 (0.97, 1.01)	0.6965
Female	0.99 (0.98, 1.01)	
Age		
< 44	1.00 (0.98, 1.02)	0.4286
44 ≤ Age < 60	0.99 (0.98, 1.01)	
≥ 60	0.98 (0.96, 1.00)	
Race		
Non‐Hispanic white	0.99 (0.97, 1.00)	0.3751
Non‐Hispanic black	1.01 (0.98, 1.04)	
Mexican American	1.01 (0.98, 1.03)	
Others	0.98 (0.96, 1.01)	
Diabetes		
Yes	0.98 (0.95, 1.00)	0.3186
No	1.00 (0.98, 1.01)	
Borderline	1.00 (0.93, 1.06)	
CVD		
Yes	1.00 (0.98, 1.01)	0.1896
No	0.99 (0.97, 1.00)	
Hepatitis B		
Yes	1.19 (0.00, inf.)	0.9986
No	0.99 (0.98, 1.00)	
CSF		
Yes	1.00 (0.98, 1.02)	0.2756
No	0.99 (0.97, 1.00)	

Abbreviation: Inf, An infinite value that exceeds the range a computer can represent.

## 5 Discussion

In this nationally representative cross‐sectional study, we observed an inverse association between niacin intake and depression among MAFLD patients in both the unadjusted (crude) and minimally adjusted models. However, this association became nonsignificant in the fully adjusted model. This may be attributable to the inclusion of additional highly correlated covariates in the fully adjusted model, which resulted in wider confidence intervals and reduced statistical power (Schisterman et al. 2017). Further analysis using smooth curves revealed a nonlinear relationship, with a breakpoint at approximately 10.24 mg/day of niacin intake. Below this threshold, niacin intake was negatively associated with depression risk, whereas no significant association was observed above the threshold. Subgroup analyses showed consistent effects across all stratified groups (gender, age, race, diabetes status, CVD, and hepatitis B; all *p* for interaction > 0.05), demonstrating the robustness of the results.

To our knowledge, this is the first study to investigate the association between niacin intake and depression risk in individuals with MAFLD. However, previous studies have separately explored the relationships of niacin with fatty liver disease and with depression. Existing evidence suggests a beneficial association between niacin and fatty liver disease. Preclinical studies have demonstrated niacin's ability to inhibit and reverse hepatic steatosis and inflammation (Hu et al. 2012; Pirinen et al. 2020). Cross‐sectional clinical evidence further indicates that higher dietary niacin intake is associated with lower liver fat content and reduced NAFLD prevalence (Linder et al. 2019; Pan et al. 2023). Additionally, increased niacin intake may lower all‐cause mortality risk in NAFLD patients (Pan et al. 2024). However, while the niacin–fatty liver relationship has been extensively studied in NAFLD, research focusing specifically on MAFLD remains limited.

The association between niacin and depression has been documented since the 1950s, with case reports suggesting that niacin supplementation may alleviate depression and anxiety symptoms (Thompson and Proctor 1953). More recently, NHANES studies revealed a *U*‐shaped relationship between niacin intake and depression risk, with varying reference intake levels (Tian et al. 2023; Zhao et al. 2023). A Korean study found an inverse association between niacin intake and depression (Nguyen et al. 2022), whereas a UK cohort study found no long‐term effect of niacin intake on psychological distress in adults (Mishra et al. 2009). These divergent findings indicate a potentially complex or contradictory association between niacin and depression.

Although niacin has been linked to improved fatty liver in numerous studies, its association with depression remains unclear. To address this gap, we investigated the relationship between niacin intake and depression in the MAFLD population. After adjusting for confounders as much as possible, we identified a nonlinear relationship between niacin intake and depression in individuals with MAFLD. Notably, below the breakpoint of approximately 10.24 mg/day, a negative correlation was observed, suggesting that niacin intake is associated with reduced depression risk in the MAFLD population.

Patients with depression often exhibit elevated levels of inflammatory cytokines and C‐reactive protein. The primary pathophysiological mechanisms underlying the interaction between niacin intake and depression in MAFLD may involve both inflammatory and lipid pathways (Miller 2020). Niacin has been shown to downregulate plasma levels of inflammatory factors and inhibit vascular inflammation in both in vivo and in vitro studies (Ibrahim et al. 2022). Furthermore, niacin might improve depression by reducing oxidative stress in endothelial cells through increased NADP levels, decreased glutathione, and suppressed production of reactive oxygen species (Ganji et al. 2009). Since excessive oxidative stress and reduced hepatic NAD levels are also characteristic of fatty liver development, niacin could simultaneously improve depression and inhibit hepatic steatosis. Additionally, niacin may modulate plasma lipids by upregulating factors involved in reverse cholesterol transport, increasing HDL‐associated proteins, blocking inflammatory cytokines, and reducing depressive symptoms (Ronsein et al. 2021)—effects that might similarly benefit fat deposition in MAFLD patients. The gut–liver–brain axis hypothesis could provide a plausible explanation, as certain probiotics have been shown to reduce lipid markers in obese patients, modulate hepatic metabolite synthesis, and exert multifaceted effects on cognition, emotion, and the nervous system (Yan et al. 2023). These proposed mechanisms are speculative, based on existing literature, and require further experimental validation.

Although the exact mechanisms underlying the negative association between niacin intake and depression in MAFLD require further investigation, our findings are biologically plausible based on available evidence. We identified a reference threshold of approximately 10.24 mg/day, suggesting that MAFLD patients might experience improved depressive symptoms with daily niacin intake around this level. This dose is relatively low compared to previous studies, a discrepancy that may be explained by the fact that MAFLD patients are typically obese, consume excessive calories, and have high intakes of other nutrients that could modulate the effects of dietary niacin on depression.

Furthermore, niacin from dietary sources and niacin from supplements may exert different physiological effects, primarily due to differences in absorption efficiency and bioavailability. Supplemental niacin typically exists in a free form, allowing more direct and efficient absorption compared to dietary niacin. Moreover, dietary niacin is generally consumed at lower doses, whereas supplemental niacin can reach pharmacological levels. At high doses, supplemental niacin may undergo distinct catabolic pathways, producing different metabolite profiles—for example, causing skin flushing, a reaction rarely observed with dietary niacin (Institute of Medicine Standing Committee on the Scientific Evaluation of Dietary Reference, its Panel on Folate, and Choline 1998). Consequently, dietary sources are suitable for long‐term, balanced intake, while supplements are more appropriate when a rapid increase in NAD^+^ levels is desired. Despite these differences, both sources ultimately contribute to the body's niacin status and share key metabolic pathways, including the synthesis of NAD^+^ (MacKay et al. 2012). From a nutritional epidemiology perspective, combining the two into a single measure better reflects the overall exposure of total niacin intake in relation to health outcomes.

Our study has several notable strengths. First, the large sample size enhances the credibility and comprehensiveness of our findings. Second, we incorporated dietary supplement data when calculating niacin intake, improving the accuracy and reliability of our exposure assessment. Third, we performed extensive subgroup analyses and interaction tests to verify the robustness of our results. Most significantly, as the first study to examine the niacin–depression relationship in the newly defined MAFLD population, our work provides novel insights distinct from previous NAFLD‐focused research. However, this study has several limitations. First, the cross‐sectional design limits causal inference between niacin intake and depression in MAFLD patients. Second, despite adjusting for numerous important covariates, we could not fully eliminate the influence of other potential confounding variables. Third, although we implemented appropriate methods to handle missing covariate data, the potential impact of missing values cannot be completely eliminated. Finally, reliance on self‐reported dietary questionnaires to assess niacin intake may introduce recall bias, potentially affecting the accuracy of our exposure measurements. Therefore, our results should be interpreted with appropriate caution.

## 6 Conclusion

Moderate niacin intake is associated with a lower risk of depression in patients with MAFLD. An approximate threshold of 10.24 mg/day was identified as a reference for future research. Randomized controlled trials are warranted to confirm these findings.

## Author Contributions

**Yan Chen:** writing – review and editing, funding acquisition. **Xiaoxian Yang:** conceptualization, methodology, writing – original draft, data curation. **Haiyi Yan:** data curation. **Xueying Qin:** data curation.

## Funding

This study was supported by Xiyuan Hospital CACMS Enhancement Fund XYZX0101‐04.

## Ethics Statement

This is an observational study. The studies involving human participants were reviewed and approved by the NCHS Ethics Review Board.

## Consent

The participants provided their written informed consent to participate in this study.

## Conflicts of Interest

The authors declare no conflicts of interest.

## Data Availability

This cross‐sectional study analyzes publicly available datasets from the National Health and Nutrition Examination Survey (NHANES) dataset, which can be available at: www.cdc.gov/nchs/nhanes/.
